# Effects of laparoscopic distal gastrectomy with Roux-en-Y anastomosis on postoperative gastrointestinal symptom recovery and bile reflux incidence in patients with gastric cancer

**DOI:** 10.3389/fonc.2026.1838556

**Published:** 2026-08-05

**Authors:** Yong Ruan, Ying Yang, Jinghong Yu, Bin Zhang

**Affiliations:** 1 Department of Colorectal and Anal Surgery, The Second Hospital of Huangshi, Huangshi, Hubei, China; 2 Department of Public Health, The Second Hospital of Huangshi, Huangshi, Hubei, China

**Keywords:** bile reflux, Billroth-II anastomosis, gastrojejunostomy, laparoscopic distal gastrectomy, Roux-en-Y anastomosis, stomach neoplasms

## Abstract

**Background:**

The optimal reconstruction method after laparoscopic distal gastrectomy (LDG) for gastric cancer is debated. This study compared Billroth-II (BII) and Roux-en-Y (RY) anastomoses in terms of postoperative recovery, bile reflux, and quality of life.

**Methods:**

This retrospective cohort study enrolled patients with gastric cancer undergoing LDG from June 2022 to June 2024. These patients were stratified into the LDG-BII and LDG-RY groups. Data collected included perioperative outcomes, gastric pH measured preoperatively and on postoperative days 1–3, gastrointestinal symptoms assessed employing the Patient Assessment of Gastrointestinal Symptoms (PAGI-SYM) questionnaire at six months postoperation, endoscopic findings evaluated by using the Ruggeri–Giustini–Balestra classification at six months postoperation, complications, and nutritional and quality of life assessments obtained with the European Organization for Research and Treatment of Cancer Quality of Life Questionnaire-Stomach Module at six months postoperation.

**Results:**

The analysis included 257 patients (LDG-BII: 126; LDG-RY: 131). Compared with the LDG-BII group, the LDG-RY group had longer anastomosis times (30.24 min vs. 28.89 min, P < 0.001) but earlier first flatus (P = 0.003) and shorter hospital stay (11.28 vs. 12.17 days, P = 0.027). The LDG-BII group had significantly higher gastric pH on postoperative day 1 (6.91 vs. 6.58, P < 0.001), worse six-month reflux symptoms (PAGI-SYM score: 14.15 vs. 12.71, P < 0.001), and higher rates of substantial endoscopic bile reflux (grade 2: 17.46% vs. 6.11%, P < 0.001) than the LDG-RY group. Quality of life related to reflux was worse in the LDG-BII group (gastric cancer–specific quality of life questionnaire score: 3.57 vs. 3.12, P < 0.001) than in the LDG-RY group.

**Conclusion:**

Compared with BII, RY reconstruction after LDG reduces bile reflux and improves key patient-reported and endoscopic outcomes despite having a longer operative time.

## 1 Introduction

Gastric cancer is a global health burden characterized by the malignant proliferation of cells within the stomach wall (1). Surgical resection, primarily gastrectomy, is the cornerstone of treatment for localized disease (2). In recent years, laparoscopic distal gastrectomy (LDG) has become the preferred surgical method because of its minimally invasive nature, which can reduce postoperative pain, shorten hospital stays, and accelerate recovery (3).

The choice of the gastrointestinal reconstruction method after distal gastrectomy is critical. Billroth II (BII) and Roux-en-Y (RY) anastomoses are two popular reconstruction methods. Although both aim to restore function and reduce postoperative complications, particularly bile reflux and associated gastrointestinal symptoms, the optimal technique to minimize these issues remains a subject of ongoing debate and a key limitation in current clinical practice (4). Bile reflux is a common complication after gastrectomy, especially with BII reconstruction, leading to residual gastritis, esophagitis, and negative effects on long-term quality of life (5, 6). RY reconstruction is thought to reduce bile reflux and improve postoperative outcomes but may increase surgical complexity and anastomosis time (7). Therefore, the optimal reconstruction method after cancer LDG remains a contentious topic, requiring further evidence to clarify its influence on postoperative gastrointestinal symptom recovery and bile reflux incidence (8).

The pathophysiological background linking the choice of the anastomosis method to clinical outcomes primarily focuses on anatomical and physiological changes. Bile reflux occurs when duodenal contents, including bile and pancreatic juice, retrograde into the gastric remnant or esophagus because of the loss of the pyloric sphincter and alterations in gastro–duodenal anatomy (9). The RY reconstruction is specifically designed to divert bile and pancreatic secretions away from the gastric remnant, theoretically reducing alkaline reflux (10). By contrast, the BII anastomosis creates a direct loop between the stomach and jejunum, increasing the likelihood of bile reflux in this segment (11, 12). This mechanistic difference is considered the basis for variations in the postoperative symptom burden and endoscopic findings of gastritis (13).

In this retrospective study, we compare the postoperative outcomes of LDG with those of BII and RY reconstruction in patients with gastric cancer. Our work combines objective physiological data, subjective symptom scores, and endoscopic assessments to provide a comprehensive comparison. Our findings aim to guide surgical decisions and optimize reconstruction techniques to improve recovery and reduce bile reflux–related morbidity.

## 2 Methods

### 2.1 Study design and ethical statement

This study is a retrospective cohort analysis. We consecutively enrolled patients with gastric cancer who underwent LDG at our hospital from June 2022 to June 2024. Patients were divided into two groups on the basis of the type of gastrointestinal reconstruction used: the LDG with BII anastomosis (LDG-BII) (n = 126) and LDG with RY anastomosis (LDG-RY)(n = 131) groups. Baseline demographic data, clinicopathological characteristics, and perioperative data were collected and compared between the two groups by systematically reviewing electronic medical records to evaluate the effect of the two anastomotic methods on postoperative recovery.

This study has been approved by the ethics review committee of our hospital. Given the retrospective nature of this study, no intervention was made in patients’ treatment decisions, and all data analyses were anonymized. The ethics committee approved the waiver of informed consent from patients. The study process strictly adhered to the principles of the Declaration of Helsinki, ensuring patient privacy and data security.

All patients in this study underwent standardized LDG. Surgeries were performed by an experienced surgical team and adhered to the principles of radical gastrectomy for gastric cancer, including D2 lymph node dissection. After completing the mobilization and resection of the stomach, gastrointestinal reconstruction was performed in accordance with the predetermined protocol.

The choice of reconstruction type was made by the attending surgeon on the basis of intraoperative assessment and surgical preference. BII and RY procedures were technically feasible in all patients. The final surgical plan was determined by the surgeon during the operation on the basis of individual anatomical considerations.

In the LDG-BII group, the gastric remnant (usually on the greater curvature side) was anastomosed to the jejunum approximately 15–20 cm distal to the Treitz ligament in an antecolic functional end-to-side manner, forming a gastrojejunostomy with a size of approximately 4–6 cm.

In the LDG-RY group, the jejunum was divided 20–25 cm distal to the Treitz ligament, and the distal jejunal stump was brought up to perform an end-to-side anastomosis with the gastric remnant. Subsequently, at approximately 40–60 cm distal to the gastrojejunal anastomosis, the previously divided proximal jejunum was anastomosed to the distal jejunum in a side-to-side manner, thereby establishing an antireflux pseudostomach pouch.

### 2.2 Inclusion and exclusion criteria

#### 2.2.1 Inclusion criteria

Patients were included if they were over 18 years old; had a pathological diagnosis of gastric cancer (14); and were confirmed intraoperatively to have no distant metastasis, such as peritoneal or liver metastasis. Patients were included if they were in good general condition with an Eastern Cooperative Oncology Group performance status score of 0–1 (15); no severe cardiopulmonary disease or other organ dysfunction that could tolerate surgical treatment; no history of other systemic malignancies; and complete clinical and pathological data.

#### 2.2.2 Exclusion criteria

Patients were excluded if any of the following conditions were present: intraoperative findings of perforation; bleeding; or tumor invasion into adjacent organs, such as the pancreas or colon. Patients with gastric obstruction, pyloric obstruction, or requirement for palliative resection were also excluded. Any patients with severe comorbidities, such as severe diabetes and chronic lung disease, or contraindications to surgery were excluded. Patients whose surgeries were terminated because of intraoperative complications, who received neoadjuvant or adjuvant chemotherapy, and with incomplete data were also excluded.

### 2.3 Data collection

#### 2.3.1 Baseline characteristics

Data on demographic and clinical characteristics, including age, gender, and BMI, were obtained from admission records. Preoperative laboratory nutritional indicators, including hemoglobin, total protein, serum albumin, and cholesterol, were collected from fasting venous blood samples taken before surgery and analyzed by using an automated biochemical analyzer (Cobas 8000, Roche Diagnostics, Switzerland). Pathological tumor staging was determined on the basis of postoperative paraffin specimen pathology reports, referring to the eighth edition of the American Joint Committee on Cancer TNM staging system (16). Stage IB corresponds to T1–T2N1 or T3N0; stage IIA corresponds to T1–T2N2 or T3N1; stage IIIB corresponds to T1–T2N3a, T3N2, or T4aN0–N1; and stage IIIA corresponds to T1–T2N3b, T3N3a, T4aN2, or T4bN0–N1.

#### 2.3.2 Perioperative outcomes

Perioperative data, including operative time, anastomosis time, intraoperative blood loss, time to first flatus, time to first oral intake, time to first ambulation, and hospital stay length, were extracted from anesthesia records, operating room records, and electronic nursing notes.

#### 2.3.3 Early postoperative pH values of gastric juice

All patients had 2 mL of gastric juice sampled through an indwelling nasogastric tube in the morning on the day before surgery and on postoperative days 1, 2, and 3. The samples were measured within 30 min of collection by using a calibrated portable pH meter (FiveGo™ F2, Mettler-Toledo International Inc., Switzerland). Prior to measurement, the instrument was calibrated by using standard buffer solutions (pH 4.01 and 7.00) at two points. Each sample was measured three times, and the average value was recorded. The procedure was performed by the same trained research nurse to ensure the consistency and accuracy of the measurements.

#### 2.3.4 Postoperative gastrointestinal symptoms

At the six-month follow-up after surgery, the Chinese version of the Patient Assessment of Gastrointestinal Symptoms was used for evaluation (17). This scale is a specific symptom assessment tool consisting of 20 items covering six dimensions, namely, reflux (seven items), nausea and vomiting (three items), early satiety (four items), bloating (two items), upper abdominal pain (two items), and lower abdominal pain (two items). Each item was scored by using a six-point Likert scale (0 indicates no symptoms, and 5 indicates extremely severe symptoms). The score for each dimension is the sum of the scores of its constituent items. High scores indicate severe symptoms.

#### 2.3.5 Endoscopic examination results

Endoscopic examinations were performed by a senior endoscopist who was blinded to the reconstruction type. At six months postoperatively, all patients underwent a standardized upper gastrointestinal endoscopy to assess the status of the remnant stomach. The Remnant Stomach–Gastritis–Bile classification was used to grade the findings observed during endoscopy (18). Residual food was graded as 0 for none, 1 for minimal, and 2 for substantial. Bile reflux was graded as 0 for none, 1 for minimal, and 2 for substantial. The extent of gastritis was graded as 0 for none, 1 for localized, and 2 for diffuse.

#### 2.3.6 Postoperative complications

Postoperative complications within 30 days were recorded. They included duodenal stump leakage, chyle leakage, ileus, anastomotic bleeding, and wound infection. All complications were graded in accordance with the Clavien–Dindo classification system.

#### 2.3.7 Postoperative nutritional status and quality of life at six months

The assessment of nutritional status at six months postoperation was based on the results of fasting venous blood tests collected during the follow-up visit at that time point. The percentage change relative to preoperative baseline values was calculated by using the formula (value at six months postoperation/preoperative baseline value) × 100% to quantify the recovery of nutritional indicators.

Quality of life at six months postoperation was assessed by using the gastric cancer–specific quality of life questionnaire developed by the European Organization for Research and Treatment of Cancer (19). This questionnaire contains 22 items covering nine symptom and functional dimensions: dysphagia (four items), stomach pain (four items), reflux (three items), eating restrictions (three items), anxiety (four items), dry mouth, taste changes, body image concerns, and hair loss. Each item is scored by using a four-point Likert scale (1 indicates “not at all,” and 4 indicates “very much”). The score for each dimension is the average of the scores of its constituent items.

### 2.4 Statistical analysis

Statistical analyses were performed by using R software (Version 4.3.3, R Foundation for Statistical Computing, Austria). Continuous variables were first tested for normality by using the Shapiro–Wilk test. Variables conforming to a normal distribution were expressed as mean ± standard deviation and compared by employing independent samples t-tests. Variables not conforming to a normal distribution were expressed as median [25th, 75th percentile] and compared by using the Mann–Whitney U test. Categorical variables were expressed as frequencies (percentages), and group comparisons were performed by using the chi-squared test or Fisher’s exact test when expected frequencies were less than 5. All statistical tests were two-tailed, and a P-value less than 0.05 was considered statistically significant.

Effect sizes with 95% confidence intervals (CIs) were calculated for all comparisons to provide a comprehensive assessment of between-group differences. For normally distributed continuous variables, the mean difference with 95% CI was reported. For nonnormally distributed continuous variables, the Hodges–Lehmann median difference with 95% CI was reported. For binary categorical variables, the odds ratio with 95% CI was reported. For variables with zero events in one group, the risk difference with 95% CI was used instead. For multicategorical variables, Cramer’s V with 95% CI (estimated via bootstrap with 1000 replicates) was reported as the effect size.

## 3 Results

### 3.1 Baseline characteristics of patients

We found no significant differences in baseline characteristics between the LDG-BII and LDG-RY groups across all assessed demographic, clinical, pathological, and biochemical parameters (all P > 0.05; **Table 1**). These results suggest that the two groups were well balanced at baseline in terms of all of the assessed clinical, pathological, and biochemical parameters.

**Table 1:** Baseline characteristics of patients.

Index	LDG-BII group (n=126)	LDG-RY group (n=131)	Effect Size (95% CI)	P
Age	55.71 ± 9.11	54.64 ± 8.93	1.07 (-1.15, 3.29)	0.341
Gender (Male/Female)	96 (76.19%)/30 (23.81%)	90 (68.70%)/41 (31.30%)	1.46 (0.84, 2.53)	0.180
BMI	21.28 ± 2.74	21.42 ± 2.61	-0.14 (-0.79, 0.51)	0.673
Pathological Tumor Stage			0.036 (0.000, 0.136)	0.846
I	42 (33.33%)	41 (31.30%)		
II	56 (44.44%)	57 (43.51%)		
III	28 (22.22%)	33 (25.19%)		
Hemoglobin (g/dL)	11.08 ± 1.69	11.25 ± 1.82	-0.17 (-0.60, 0.26)	0.455
Total Proteins (g/dL)	6.53 ± 0.62	6.47 ± 0.48	0.06 (-0.08, 0.20)	0.395
Serum Albumin (g/dL)	3.63 ± 0.34	3.58 ± 0.31	0.05 (-0.03, 0.13)	0.240
Cholesterol (mmol/L)	4.58 ± 0.39	4.62 ± 0.35	-0.04 (-0.13, 0.05)	0.361
Tumor Size (cm)	2.85 ± 1.12	2.79 ± 1.08	0.06 (-0.21, 0.33)	0.678
Tumor Location (Middle third/Lower third)	35 (27.78%)/91 (72.22%)	39 (29.77%)/92 (70.23%)	0.91 (0.54, 1.54)	0.724
Histological Differentiation (Differentiated/Undifferentiated)	48 (38.1%)/78 (61.9%)	54 (41.22%)/77 (58.78%)	0.88 (0.54, 1.43)	0.609
ASA Physical Status (I/II)	42 (33.33%)/84 (66.67%)	47 (35.88%)/84 (64.12%)	0.89 (0.54, 1.47)	0.668
Extent of Lymphadenectomy (D1+/D2)	11 (8.73%)/115 (91.27%)	13 (9.92%)/118 (90.08%)	0.87 (0.37, 2.01)	0.742
Harvested Lymph Nodes	25.42 ± 5.63	25.11 ± 6.04	0.31 (-1.10, 1.72)	0.666
Hypertension	38 (30.16%)	37 (28.24%)	1.10 (0.65, 1.85)	0.736

BMI (Body Mass Index).

### 3.2 Perioperative outcomes

In the comparison of perioperative outcomes, the LDG-RY group had significantly longer operative (P = 0.003) and anastomosis (P < 0.001) times than the LDG-BII group (**Table 2**). The time to first flatus was significantly later in the LDG-BII group than in the LDG-RY group (P = 0.003). Moreover, the length of hospital stay was significantly longer in the LDG-BII group than in the LDG-RY group (P = 0.027). We discovered no significant differences between the two groups in terms of intraoperative blood loss (P = 0.676), time to first oral intake (P = 0.515), and time to first ambulation (P = 0.061).

**Table 2:** Perioperative outcomes.

Index	LDG-BII group (n=126)	LDG-RY group (n=131)	Effect size (95% CI)	P
Operative Time (min)	178.55 ± 15.11	184.83 ± 17.84	-6.28 (-10.36, -2.20)	0.003
Anastomosis Time (min)	28.89 ± 2.84	30.24 ± 2.59	-1.35 (-2.02, -0.68)	< 0.001
Blood Loss (mL)	74.52 ± 6.36	74.18 ± 6.67	0.34 (-1.27, 1.95)	0.676
Onset of Passing Flatus (days)	3.00 [2.00, 4.00]	3.00 [2.00, 3.00]	0.00 (0.00, 0.00)	0.003
First Oral Intake (days)	4.00 [3.00, 4.00]	4.00 [3.00, 4.00]	0.00 (0.00, 0.00)	0.515
First Ambulation (days)	2.00 [1.00, 2.00]	2.00 [1.00, 2.00]	0.00 (0.00, 0.00)	0.061
Length of Hospital Stay (days)	12.17 ± 3.56	11.28 ± 2.79	0.89 (0.10, 1.68)	0.027

### 3.3 Early postoperative changes in gastric acid pH value

**Figure 1:**
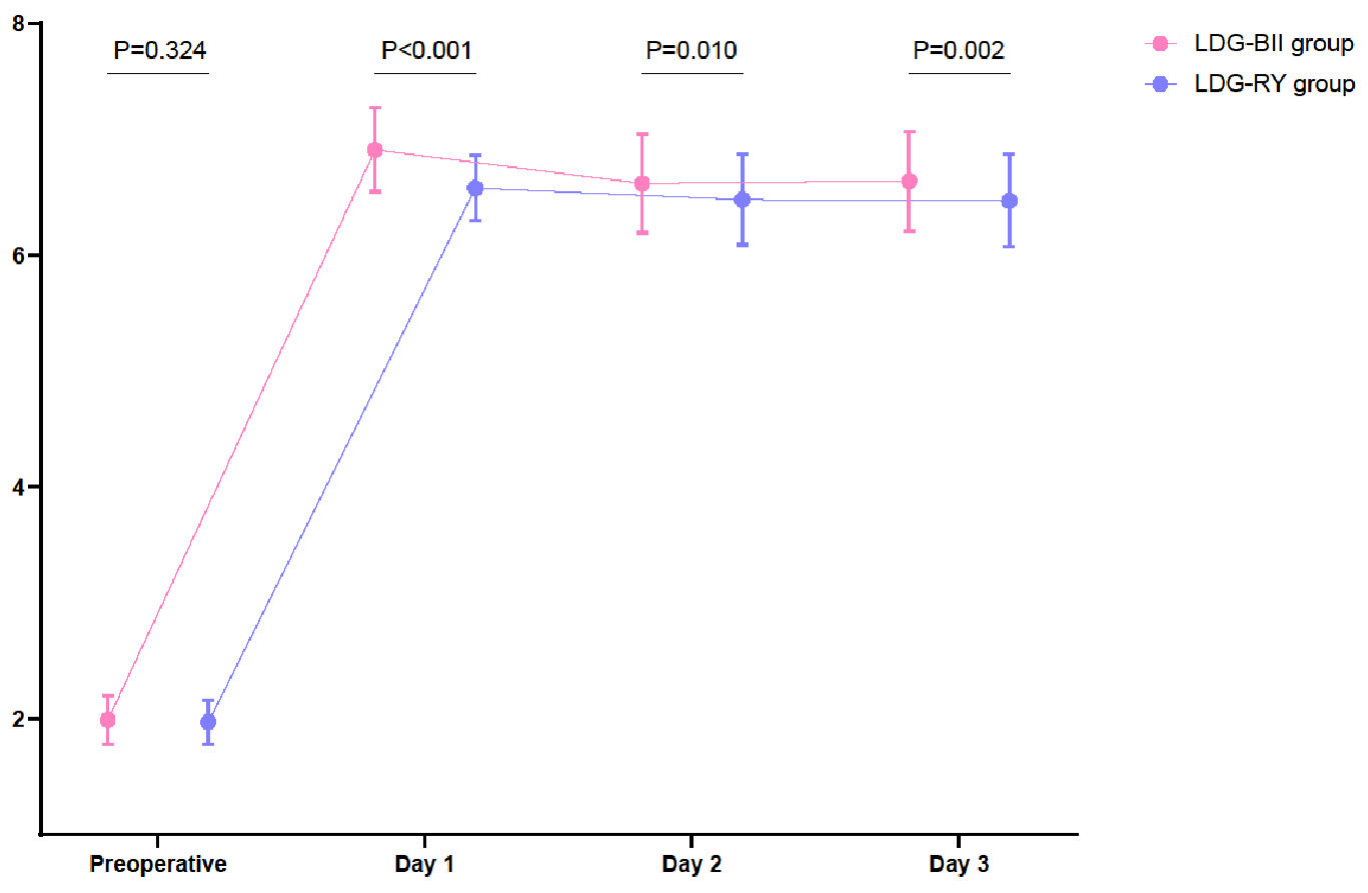
Dynamic changes in gastric pH during the early postoperative period.

We observed no significant differences in preoperative gastric pH values between the LDG-BII and LDG-RY groups (P = 0.324; **Figure 1**). The intragastric pH value on postoperative day 1 was significantly higher in the LDG-BII group than in the LDG-RY group (P < 0.001), and this significant difference persisted through postoperative days 2 (P = 0.010) and 3 (P = 0.002). These results indicate that although the two groups had similar preoperative intragastric pH values, the LDG-BII group exhibited higher intragastric pH values than the LDG-RY group during the first few days after surgery.

### 3.4 Postoperative gastrointestinal symptoms

In terms of postoperative gastrointestinal symptom scores, the reflux score was significantly higher in the LDG-BII group than in the LDG-RY group (P < 0.001). Moreover, the LDG-BII group has significantly higher nausea and vomiting (P = 0.006), early satiety and bloating (P = 0.018), and bloating (P = 0.007) scores than the LDG-RY group (**Table 3**). We found no significant differences between the two groups in terms of upper (P = 0.589) and lower (P = 0.640) abdominal pain. These results indicate that compared with those in the LDG-RY group, patients in the LDG-BII group exhibited a higher severity of symptoms, such as reflux, nausea and vomiting, early satiety, and bloating.

**Table 3:** Postoperative gastrointestinal symptom scores assessed by the PAGI-SYM questionnaire.

Index	LDG-BII group (n=126)	LDG-RY group (n=131)	Effect size (95% CI)	P
Reflux	14.15 ± 2.64	12.71 ± 2.42	1.44 (0.82, 2.06)	< 0.001
Nausea and Vomiting	4.06 ± 1.17	3.65 ± 1.13	0.41 (0.13, 0.69)	0.006
Early Satiety and Bloating	9.17 ± 1.82	8.64 ± 1.78	0.53 (0.09, 0.97)	0.018
Bloating	2.36 ± 0.55	2.19 ± 0.49	0.17 (0.04, 0.30)	0.007
Upper Abdominal Pain	1.25 ± 0.35	1.27 ± 0.37	-0.02 (-0.11, 0.07)	0.589
Lower Abdominal Pain	1.28 ± 0.33	1.26 ± 0.29	0.02 (-0.06, 0.10)	0.640

### 3.5 Endoscopic results at six months after surgery

Our endoscopic examination results at six months postoperation showed that the residual food grade was significantly higher in the LDG-BII group than in the LDG-RY group (P = 0.041), indicating that patients in the LDG-BII group had more residual food than those in the LDG-RY group (**Table 4**). The LDG-BII group showed a significantly higher incidence of bile reflux than the LDG-RY group (P < 0.001). Additionally, the degree of gastritis was significantly higher in the LDG-BII group than in the LDG-RY group (P = 0.001). These results demonstrate that the LDG-BII group exhibited more severe conditions in terms of residual food, bile reflux, and gastritis degree than the LDG-RY group.

**Table 4:** Endoscopic and histopathological findings at 6 months postoperatively.

Index	LDG-BII group (n=126)	LDG-RY group (n=131)	Effect size (95% CI)	P
Residual Food			0.16 (0.01, 0.30)	0.041
Grade 0	111 (88.10%)	126 (96.18%)		
Grade 1	13 (10.32%)	5 (3.82%)		
Grade 2	2 (1.59%)	0 (0.00%)		
Bile Reflux			0.27 (0.16, 0.38)	< 0.001
Grade 0	49 (38.89%)	81 (61.83%)		
Grade 1	55 (43.65%)	42 (32.06%)		
Grade 2	22 (17.46%)	8 (6.11%)		
Gastritis Extent			0.25 (0.11, 0.37)	0.001
Grade 0	53 (42.06%)	85 (64.89%)		
Grade 1	39 (30.95%)	26 (19.85%)		
Grade 2	34 (26.98%)	20 (15.27%)		

### 3.6 Postoperative complications

The total complication rate was significantly higher in the LDG-BII group than in the LDG-RY group (P = 0.026; **Table 5**). No individual complication category, including Clavien–Dindo I–II (P = 0.257) and III–IV (P = 0.232), reached statistical significance (all P>0.05) likely because of low event frequencies, which limit statistical power for each specific endpoint. The overall difference appears to be driven by the cumulative effect of infrequent events. This pattern suggests that BII reconstruction may be associated with a modestly increased overall complication burden.

**Table 5:** Postoperative complications.

Index	LDG-BII group (n=126)	LDG-RY group (n=131)	Effect size (95% CI)	P
Total	9 (7.14%)	2 (1.53%)	4.95 (1.05, 23.39)	0.026
Duodenal Stump Leakage	5 (3.97%)	1 (0.76%)	5.34 (0.62, 46.23)	0.198
Chylous Fistula	1 (0.79%)	0 (0.00%)	0.79% (-0.76%, 2.34%)	0.984
Ileus	1 (0.79%)	1 (0.76%)	1.04 (0.06, 16.80)	0.495
Anastomotic Bleeding	1 (0.79%)	0 (0.00%)	0.79% (-0.76%, 2.34%)	0.984
Wound Infection	1 (0.79%)	0 (0.00%)	0.79% (-0.76%, 2.34%)	0.984
Clavien-Dindo I–II	6 (4.76%)	2 (1.53%)	3.23 (0.64, 16.34)	0.257
Clavien-Dindo III–IV	3 (2.38%)	0 (0.00%)	7.13 (0.36, 139.32)	0.232

For total complications and Duodenal Stump Leakage, OR (95% CI) was calculated using the Wald method. For complications with zero events in one group (Chylous Fistula, Anastomotic Bleeding, Wound Infection), RD (95% CI) was reported with the Wilson method.

### 3.7 Nutritional status and quality of life at six months postoperation

**Figure 2:**
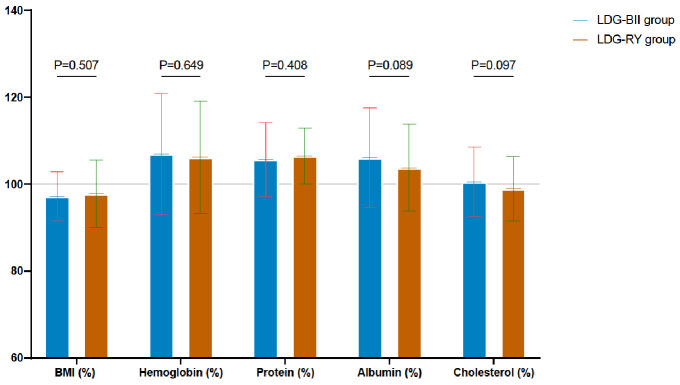
Nutritional status at 6 months postoperatively.

In the comparison of nutritional status at six months postoperation, we found no statistically significant differences between the LDG-BII and LDG-RY groups in terms of BMI and hemoglobin, protein, albumin, and cholesterol levels (all P > 0.05; **Figure 2**). These results suggest that the two surgical methods have similar effects on patients’ nutritional status.

In the quality of life assessment, we found no significant differences between the LDG-BII and LDG-RY groups in terms of dysphagia (P = 0.391), stomach pain (P = 0.102), anxiety (P = 0.235), dry mouth (P = 0.091), taste changes (P = 0.103), body image (P = 0.172), and hair loss (P = 0.188; **Table 6**). The reflux symptom score was significantly higher in the LDG-BII group than in the LDG-RY group (P < 0.001). Furthermore, the eating restriction score was significantly higher in the LDG-BII group than in the LDG-RY group (P = 0.033). These results indicate that in terms of reflux and eating restrictions, patients in the LDG-BII group reported more severe symptoms than those in the LDG-RY group, suggesting that this group may face a greater effect on their quality of life.

**Table 6:** Quality of life.

Index	LDG-BII group (n=126)	LDG-RY group (n=131)	Effect size (95% CI)	P
Dysphagia	0.58 ± 0.19	0.56 ± 0.17	0.02 (-0.03 to 0.07)	0.391
Stomach Pain	1.22 ± 0.40	1.15 ± 0.35	0.07 (-0.01 to 0.15)	0.102
Reflux	3.57 ± 0.69	3.12 ± 0.60	0.45 (0.29 to 0.61)	< 0.001
Eating Restrictions	2.35 ± 0.62	2.18 ± 0.58	0.17 (0.02 to 0.32)	0.033
Anxiety	1.38 ± 0.43	1.32 ± 0.35	0.06 (-0.04 to 0.16)	0.235
Dry Mouth	0.92 ± 0.31	0.85 ± 0.35	0.07 (-0.01 to 0.15)	0.091
Taste Changes	0.65 ± 0.35	0.59 ± 0.26	0.06 (-0.01 to 0.13)	0.103
Body Image	1.59 ± 0.89	1.46 ± 0.62	0.13 (-0.06 to 0.32)	0.172
Hair Loss	1.36 ± 0.52	1.48 ± 0.88	-0.12 (-0.30 to 0.06)	0.188

## 4 Discussion

The choice of reconstruction after distal gastrectomy is crucial for postoperative physiology and patients’ quality of life. In this study, we compared BII and RY anastomosis in laparoscopic surgery. Our findings show that while RY requires more technical complexity and longer operative time than RY, it offers advantages in reducing alkaline and bile reflux, improving gastrointestinal symptoms, and enhancing endoscopic and quality of life outcomes in the medium term.

The long operative and anastomotic times observed in the RY group are consistent with the findings reported in previous surgical literature. This increased duration, which stems from the need to construct two anastomoses and divide the mesentery, represents a well-recognized technical trade-off (20). Nevertheless, this prolonged operative time did not translate into poor early recovery; on the contrary, the RY group demonstrated an earlier return of bowel function and shorter hospital stays than the BII group. This improvement may be attributable to the highly effective bile and pancreatic secretion diversion that reduces local inflammation and alleviates bowel obstruction (21). This mechanistic interpretation is supported by studies showing that diverting bile away from the gastric remnant can mitigate inflammation and enhance gastric motility (22). Recent network meta-analyses have similarly concluded that compared with BII anastomosis, RY reconstruction, despite its longer operative time, is associated with the faster recovery of gastrointestinal function (23). Our findings confirm that RY’s functional benefits can enhance early postoperative recovery, offsetting the initial time investment.

Postoperative gastric pH changes provide physiological evidence for the antireflux design of RY reconstruction. Within three days, the BII group showed increased pH, indicating increased alkaline reflux in the remnant stomach due to the direct entry of duodenal contents (24). By contrast, the 40–60 cm Roux limb in RY acts as a barrier, diverting these secretions (25). This early pH difference can lead to later clinical issues, such as remnant gastritis. Previous studies confirmed reduced duodeno–gastric reflux in RY, setting the stage for a reduced number of subsequent symptoms (26). These findings are consistent with the results of a large meta-analysis, which demonstrated that BII with Braun anastomosis was associated with a significantly higher risk of bile reflux compared with RY reconstruction (27).

Symptom assessment at six months postoperation showed that RY reconstruction offers clear advantages in alleviating reflux-related distress. Patients who underwent BII reconstruction reported high severity across multiple domains, including reflux, nausea, vomiting, early satiety, and bloating. This result is directly related to the burden of bile reflux and its effect on gastric motility and sensory function. Alkaline reflux gastritis can cause mucosal edema and inflammation, leading to symptoms, such as upper abdominal discomfort, bloating, and early satiety (28). Bile reflux into the lower esophagus can also lead to heartburn-like symptoms (29). Consistent with the findings of previous studies reporting good symptom scores after RY reconstruction, the creation of a pseudostomach pouch and ability to divert bile in RY reconstruction alleviate these symptoms (30). The consistency between our symptom findings and pH value data strengthens a coherent pathophysiological narrative. These results resonate with the findings of several prospective and randomized studies that have established RY reconstruction as superior to BII in reducing specific postgastrectomy syndromes, particularly those related to reflux (31).

Endoscopic evaluation further corroborated the symptom findings, with the BII group showing a higher prevalence of residual food, bile reflux, and gastritis than the RY group. The substantial residual food present in the remnant stomach can become a breeding ground for bacterial overgrowth and chronic inflammation, potentially exacerbating gastritis and leading to cyclical reflux symptoms (27). The increased bile reflux observed in the BII cohort is a direct result of the anatomical continuity between the stomach and jejunum, allowing duodenal contents to flow retrograde (32). This biliary gastritis not only underpins the endoscopic findings but also provides a plausible mechanism linking the increased reflux and dyspepsia symptoms reported by these patients. The lower levels of bile reflux and gastritis in the RY group than in the BII group highlight the protective role of the Roux limb in safeguarding mucosal health and reducing inflammatory sequelae. Our findings are highly consistent with the extensive endoscopic literature that consistently identifies BII anastomosis as a major risk factor for severe remnant gastritis and bile reflux.

Postoperative complications were higher in the BII group than in the RY group. This difference is plausibly related to the anatomical configuration of BII reconstruction. In BII anatomy, the closed duodenal stump is predisposed to elevated intraluminal pressure because of afferent loop stasis or impaired drainage, which may compromise suture integrity (33). By contrast, RY reconstruction diverts biliary and pancreatic secretions away from the duodenal stump, potentially reducing intraluminal pressure and reducing the risk of stump-related complications (34). While meta-analyses have shown comparable overall morbidity between the two techniques, the cumulative effect of the infrequent events observed in this study suggests a modestly increased complication burden associated with BII reconstruction (35).

The nutritional status of both groups was similar at six months, indicating that both reconstruction methods support adequate recovery. The antireflux benefits of the RY method are achieved without compromising the body’s ability to absorb macronutrients or maintain protein stores. Similar nutritional outcomes suggest that despite differences in symptoms and endoscopic findings, both techniques meet postoperative physiological needs (36).

Regarding quality of life, the RY group scored better in terms of reflux and dietary restrictions than the BII group. These results are consistent with our symptom and endoscopic findings. The above issues stem from bile reflux and residual food, which can impair meal enjoyment and lead to restrictive eating (37). No differences in pain, anxiety, or body image were observed, suggesting that the core benefits of RY are limited to reflux-related improvements without introducing new harm.

While our study provides valuable insights, several limitations warrant consideration. The retrospective nature of our analysis introduces potential selection bias because the choice of the reconstruction method may have been influenced by surgeon preferences or intraoperative findings not captured in the dataset. Propensity score matching was not performed given the limited sample size, which would have resulted in a substantial loss of statistical power for detecting differences in primary outcomes, such as bile reflux. However, the baseline comparison demonstrated that the two groups were well balanced across all measured variables, partially mitigating this concern. Additionally, the single-center design of our work limits the generalizability of our findings to different surgical settings and patient populations. The six-month follow-up period is appropriate for assessing early symptom recovery and endoscopic bile reflux, which are the primary endpoints of our study, but is insufficient for drawing definitive conclusions regarding long-term nutritional status. The nutritional parameters evaluated at six months should therefore be interpreted as intermediate-term recovery indicators. Future prospective multicenter trials with extended follow-up periods are essential to validate our findings and explore the longitudinal effect of reconstruction choices on survival, recurrence, and sustained quality of life.

## 5 Conclusion

Compared with BII anastomosis, RY anastomosis is associated with less bile reflux after LDG, leading to improved gastrointestinal symptoms and endoscopic gastric mucosa status in patients. Although it has a slightly longer operative time than BII anastomosis, RY anastomosis results in improved postoperative recovery and quality of life.

## Data Availability

The raw data supporting the conclusions of this article will be made available by the authors, without undue reservation.

## References

[1] Bray F Laversanne M Sung H Ferlay J Siegel RL Soerjomataram I . Global cancer statistics 2022: GLOBOCAN estimates of incidence and mortality worldwide for 36 cancers in 185 countries. CA Cancer J Clin. (2024) 74:229–63. doi: 10.3322/caac.21834 38572751

[2] Takahashi C Glasser J Schuster C Huston J Shridhar R Meredith K . Comparative outcomes of laparoscopic and robotic approaches to gastrectomy: a National Cancer Database study. Surg Endosc. (2023) 37:7530–7. doi: 10.1007/s00464-023-10204-9 37433916

[3] Kang SH Yoo M Hwang D Lee E Lee S Park YS . Postoperative pain and quality of life after single-incision distal gastrectomy versus multiport laparoscopic distal gastrectomy for early gastric cancer - a randomized controlled trial. Surg Endosc. (2023) 37:2095–103. doi: 10.1007/s00464-022-09709-6 36307602 PMC9616415

[4] Seo H Son SY Hur H Park YS Han DS Lee HH . Reconstruction after distal gastrectomy and alkaline gastritis: a multi-center three-arm randomized trial comparing Billroth-II, Billroth-II Braun, and Uncut Roux-en-Y (SDR-02). Int J Surg. (2025) 112:7160–71. doi: 10.1097/js9.0000000000004490 41417990

[5] Kim TH Park JH Jeong SH Cha RR Kim HJ Lee YJ . Comparative analysis of endoscopic and nutritional outcomes following distal gastrectomy: Roux-en-Y, uncut Roux-en-Y, and Billroth II with Braun anastomosis. Korean J Clin Oncol. (2025) 21:121–9. doi: 10.14216/kjco.25364 41508661 PMC12784151

[6] Christodoulidis G Kouliou MN Koumarelas KE Argyriou K Karali GA Tepetes K . Billroth II anastomosis combined with brown anastomosis reduce reflux gastritis in gastric cancer patients. World J Methodol. (2024) 14:89709. doi: 10.5662/wjm.v14.i1.89709 38577202 PMC10989415

[7] Mikami S Enomoto T Shimada J Hisatsune Y Tsukamoto Y Hiwatari M . Evaluation of the efficacy and safety of the augmented rectangle technique for Billroth-I reconstruction in laparoscopic distal gastrectomy for gastric cancer: comparison with Roux-en-Y reconstruction. Surg Endosc. (2026) 40:192–204. doi: 10.1007/s00464-025-12281-4 41053382 PMC12823624

[8] Chen YX Huang QZ Wang PC Zhu YJ Chen LQ Wu CY . Short- and long-term outcomes of Roux-en-Y and Billroth II with Braun reconstruction in total laparoscopic distal gastrectomy: a retrospective analysis. World J Surg Oncol. (2023) 21:361. doi: 10.1186/s12957-023-03249-6 37990273 PMC10664253

[9] Zhao B Yu Z Hu T . Comparative efficacy of uncut Roux-en-Y and Billroth II anastomosis in gastrointestinal reconstruction following laparoscopic radical gastrectomy for distal gastric cancer. Med (Baltimore). (2024) 103:e37037. doi: 10.1097/md.0000000000037037 38306517 PMC10843378

[10] Yu J Li M Qin XZ Gong L Qin L Lv ZB . Application of modified Roux-en-Y digestive tract reconstruction in total gastrectomy for patients with gastric cancer. World J Gastrointest Surg. (2025) 17:106009. doi: 10.4240/wjgs.v17.i6.106009 40584499 PMC12188575

[11] Cosma CD Butiurca VO Botoncea M Nicolescu C Russu C Molnar C . Short- and mid-term surgical outcomes of Billroth I versus Billroth II/Roux-en-Y reconstruction: a prospective observational cohort study. Med (Kaunas). (2025) 61:1927. doi: 10.3390/medicina61111927 41303764 PMC12654314

[12] Lafci A Sahap M Erdem G Odemis B . Comparison of conscious and deep sedation methods in terms of pulmonary complications in ERCP procedures of patients with Billroth II gastrectomy: a retrospective study. J Clin Med. (2025) 14:5099. doi: 10.3390/jcm14145099 40725791 PMC12294892

[13] Sargsyan N Ali I Namgoong C Das B Fehervari M Fadel MG . Gastro-oesophageal reflux disease outcomes following Roux-en-Y gastric bypass surgery in patients with obesity: a systematic review and meta-analysis. Obes Surg. (2025) 35:2321–32. doi: 10.1007/s11695-025-07865-x 40272754 PMC12130072

[14] Ajani JA D'Amico TA Bentrem DJ Chao J Cooke D Corvera C . Gastric cancer, version 2.2022, NCCN clinical practice guidelines in oncology. J Natl Compr Canc Netw. (2022) 20:167–92. doi: 10.6004/jnccn.2022.0008 35130500

[15] Park SH Shin YR Hur H Lee CM Min JS Ryu SW . Exploring ideal operative time for best outcomes in gastric cancer surgery: a multi-institutional study based on KLASS-07 database. Chin J Cancer Res. (2023) 35:660–74. doi: 10.21147/j.issn.1000-9604.2023.06.10 38204442 PMC10774136

[16] Amin MB Greene FL Edge SB Compton CC Gershenwald JE Brookland RK . The eighth edition AJCC cancer staging manual: continuing to build a bridge from a population-based to a more "personalized" approach to cancer staging. CA Cancer J Clin. (2017) 67:93–9. doi: 10.3322/caac.21388 28094848

[17] Rentz AM Kahrilas P Stanghellini V Tack J Talley NJ de la Loge C . Development and psychometric evaluation of the patient assessment of upper gastrointestinal symptom severity index (PAGI-SYM) in patients with upper gastrointestinal disorders. Qual Life Res. (2004) 13:1737–49. doi: 10.1007/s11136-004-9567-x 15651544

[18] Kubo M Sasako M Gotoda T Ono H Fujishiro M Saito D . Endoscopic evaluation of the remnant stomach after gastrectomy: proposal for a new classification. Gastric Cancer. (2002) 5:83–9. doi: 10.1007/s101200200014 12111583

[19] de la Loge C Trudeau E Marquis P Kahrilas P Stanghellini V Talley NJ . Cross-cultural development and validation of a patient self-administered questionnaire to assess quality of life in upper gastrointestinal disorders: the PAGI-QOL. Qual Life Res. (2004) 13:1751–62. doi: 10.1007/s11136-004-8751-3 15651545

[20] Nabekura T Oshiro T Wakamatsu K Kitahara N Moriyama Y Kadoya K . Short-term operative outcomes of loop reconstruction for duodenojejunal bypass with sleeve gastrectomy: a retrospective study from a single Japanese academic hospital. Asian J Endosc Surg. (2026) 19:e70245. doi: 10.1111/ases.70245 41572472 PMC13349597

[21] Yang K Zhang W Chen Z Chen X Liu K Zhao L . Comparison of long-term quality of life between Billroth-I and Roux-en-Y anastomosis after distal gastrectomy for gastric cancer: a randomized controlled trial. Chin Med J (Engl). (2023) 136:1074–81. doi: 10.1097/cm9.0000000000002602 37014767 PMC10228481

[22] Abu-Zeid EED Garzali IU Aloun A Sheshe AA . Isolated Roux-en-Y versus single loop pancreaticojejunal reconstruction after pancreaticoduodenectomy - a systematic review and meta-analysis of randomised controlled trials. S Afr J Surg. (2024) 62:28–32. doi: 10.36303/sajs.00257 38838116

[23] Mirza W Khan F Khan ME Khan HM Dadan S Badshah MB . Roux-en-Y versus Billroth II reconstruction following pancreaticoduodenectomy or distal gastrectomy: a systematic review and meta-analysis of randomized controlled trials evaluating delayed gastric emptying and postoperative outcomes. Dig Dis Sci. (2026) 71:1091–107. doi: 10.1007/s10620-025-09466-1 41087609

[24] Konishi S Manaka D Morioka M Ikeda A Kudo R Saito Y . Laparoscopic-assisted robotic distal gastrectomy for gastric cancer by Billroth II reconstruction. Langenbecks Arch Surg. (2023) 408:179. doi: 10.1007/s00423-023-02895-4 37145178

[25] Moriyama T Ohuchida K Ohtsuka T Shindo K Ikenaga N Nakata K . Higher incidence of cholelithiasis with Roux-en-Y reconstruction compared with Billroth-I after laparoscopic distal gastrectomy for gastric cancer: a retrospective cohort study. Langenbecks Arch Surg. (2024) 409:75. doi: 10.1007/s00423-024-03267-2 38409456

[26] Li X Wei Q Ma J Chen L Chen M Wei C . Comparing quality of life for Billroth II with Braun versus uncut Roux-en-Y reconstruction after total laparoscopic distal gastrectomy for gastric cancer: a randomized clinical trial. Cancer Control. (2025) 32:10732748251384366. doi: 10.1177/10732748251384366 41042207 PMC12495203

[27] Park SH Hur H Park JH Lee CM Son YG Jung MR . Reappraisal of optimal reconstruction after distal gastrectomy - a study based on the KLASS-07 database. Int J Surg. (2024) 110:32–44. doi: 10.1097/js9.0000000000000796 37755373 PMC10793744

[28] Livzan MA Mozgovoi SI Gaus OV Bordin DS Kononov AV . Diagnostic principles for chronic gastritis associated with duodenogastric reflux. Diagnostics (Basel). (2023) 13:186. doi: 10.3390/diagnostics13020186 36672996 PMC9858268

[29] Iwaya Y Goda K Kako S Hattori H Miyazawa T Hara D . Association between endoscopic evidence of bile reflux and Barrett's esophagus: a large-scale case-control study. Dig Liver Dis. (2024) 56:622–7. doi: 10.1016/j.dld.2023.11.042 38105146

[30] Kubota A Yamauchi S Yoshimoto Y Tsuda K Yube Y Kaji S . Impact of the aboral pouch in Roux-en-Y reconstruction after laparoscopic total gastrectomy for elderly patients. Juntendo Iji Zasshi. (2024) 70:204–13. doi: 10.14789/jmj.jmj23-0036-oa 39429689 PMC11487364

[31] Xu H Yang L Zhang DC Li Z Li QY Wang LJ . To cut or not to cut? A prospective randomized controlled trial on short-term outcomes of the uncut Roux-en-Y reconstruction for gastric cancer. Surg Endosc. (2023) 37:6172–84. doi: 10.1007/s00464-023-10067-0 37160808 PMC10338403

[32] Lee KH Yim GH Han J Jeong HT . Clinical outcomes of endoscopic retrograde cholangiopancreatography after Billroth II anastomosis: a comparison of gastroscope and duodenoscope. BMC Gastroenterol. (2025) 25:373. doi: 10.1186/s12876-025-03973-1 40375138 PMC12079895

[33] Wang Q Wang Z Jin S Ju Y Sun P Wei Y . Double half purse-string sutures plus "8" pattern of stitching for prevention of duodenal stump fistula after laparoscopic gastrectomy. J Laparoendosc Adv Surg Tech A. (2024) 34:814–21. doi: 10.1089/lap.2024.0113 38808528

[34] Abe T Fujiya K Terashima M Notsu A Matsumoto Y Koseki Y . Risk factors for duodenal stump leakage in gastric cancer surgery and the preventive effect of duodenal stump reinforcement. World J Surg. (2025) 49:1591–9. doi: 10.1002/wjs.12610 40346729

[35] Ochoa G Marino C Dib M Briceño E Martinez JA Jarufe N . Roux-en-Y biliary reconstruction as a definitive treatment for serious complications of portal biliopathy. Case series. Int J Surg Case Rep. (2023) 110:108571. doi: 10.1016/j.ijscr.2023.108571 37574629 PMC10448269

[36] Cosma C Butiurca VO Nicolescu C Russu PC Botoncea M Molnar C . Comparative analysis of nutritional and immune status using the Conut score in patients undergoing Billroth I and Billroth II / Roux-en-Y reconstruction. Chirurgia (Bucur). (2025) 120:566–74. doi: 10.21614/chirurgia.3218 41196083

[37] Cai Z Mu M Ma Q Liu C Jiang Z Liu B . Uncut Roux-en-Y reconstruction after distal gastrectomy for gastric cancer. Cochrane Database Syst Rev. (2024) 2:Cd015014. doi: 10.1002/14651858.cd015014 38421211 PMC10903295

